# Stimuli-Responsive Polymer Systems—Recent Manufacturing Techniques and Applications

**DOI:** 10.3390/ma12152380

**Published:** 2019-07-26

**Authors:** Akif Kaynak, Ali Zolfagharian

**Affiliations:** School of Engineering, Deakin University, Geelong, Victoria 3216, Australia

**Keywords:** stimuli-responsive polymer, soft robotic actuators, 3D printing, 4D printing

Stimuli-responsive polymer systems can be defined as functional materials that show physical or chemical property changes in response to external stimuli, such as temperature, radiation, chemical agents, pH, mechanical stress, and electric and magnetic fields. Recent developments in manufacturing techniques facilitated the production of different types of stimuli-responsive polymer systems, such as micro- and nanoscale structures with potential applications in soft sensors and actuators, smart textiles, soft robots, and artificial muscles. This special issue presents one review and four scientific report articles. In the review article [1], Wang’s group from Key Laboratory of Textile Fiber and Product in Wuhan Textile University evaluates the requirements and characteristics of silk fibroin (SF) as a three-dimensional (3D) printing bioink in biomedical applications. The current challenges of cell-loading SF-based bioinks are comprehensively viewed from their physical properties, chemical components, and bioactivities. The article provides an overview of the programmable and multiple processes involved, including suggestions for further improvement of silk-based biomaterials fabrication by 3D printing. Hu’s group from Beijing University of Chemical Technology presents a paper on the preparation and processing of novel polymer materials to develop a shape memory rubber composite with a tailorable transition temperature and excellent shape recovery and fixity [2]. The proposed approach of adjusting the transition temperature of responsive rubber composites enables new design possibilities in stimuli-responsive polymer systems. 

Zolfagharian’s group from the School of Engineering in Deakin University demonstrates the applications of stimuli-responsive polymers, particularly polyelectrolyte hydrogels, in a soft robotic actuator, which is developed by 3D printing technology [3]. Due to parametric uncertainties of such actuators, which originate from both the custom-design nature of 3D printing and the time variant characteristics of polyelectrolyte actuators, a sophisticated model to estimate their behavior is developed. A practical system identification-based modeling approach for the deflection of the 3D-printed soft actuators incorporating Takagi–Sugeno (T–S) fuzzy sets is proposed and successfully tested in response to a broad range of input voltage variations. With some modifications in the electromechanical aspects of the model, the proposed modeling method can be used with other 3D-printed stimuli-responsive polymer systems. In the fourth article, Calixto’s group presents the application of stimuli-responsive materials in electronic devices to measure Relative Humidity (RH) [4]. Gelatin and interpenetrated polymers are utilized to develop an RH detector with a spark-free optical method. The water vapor is used as a stimulus to change film thickness and its refractive index. To detect the change of these two parameters, an optical method based on diffraction gratings is employed.

The special issue closes with the application of stimuli-responsive polymer systems in four-dimensional (4D) printing. Bodaghi’s group from the Department of Engineering in Nottingham Trent University presents the emergence of 4D-printed self-morphing structures manufactured by stimuli-responsive and shape memory polymers [5]. The article discusses harnessing complex structures with self-bending/morphing/rolling features fabricated by 4D printing technology, and replicate their thermo-mechanical behaviors using a simple computational tool. Fused deposition modeling (FDM) is implemented to fabricate adaptive composite structures with performance-driven functionality built directly into materials. The effects of printing speed on the self-bending/morphing characteristics are investigated in detail. Thermo-mechanical behaviors of the 4D-printed structures are simulated by introducing a straightforward method into the commercial finite element (FE) software package of Abaqus, which is much simpler than writing a user-defined material subroutine or an in-house FE code. Finally, the developed digital tool is implemented to engineer several practical self-morphing/rolling structures.
